# Clinical and Echocardiographic Determinants of Moderate Heart Failure in Children with Acyanotic Congenital Heart Disease: A Retrospective Single-Center Exploratory Prediction Modelling Study

**DOI:** 10.3390/children13060809

**Published:** 2026-06-12

**Authors:** I Ketut Alit Utamayasa, Prima Hari Nastiti, Ayurveda Zaynabila Heriqbaldi, Bagas Triambodo, Mahrus Abdur Rahman

**Affiliations:** 1 Department of Child Health, Faculty of Medicine, Universitas Airlangga, Surabaya 60132, Indonesia; prima.hari.nastiti-2019@fk.unair.ac.id (P.H.N.); bagas.triambodo-2025@fk.unair.ac.id (B.T.); mahrus.a@fk.unair.ac.id (M.A.R.); 2 Division of Pediatric Cardiology, Department of Child Health, Dr. Soetomo General Hospital, Surabaya 60286, Indonesia; 3 Faculty of Medicine, Universitas Airlangga, Surabaya 60132, Indonesia; hana.zaynabila@gmail.com

**Keywords:** congenital heart disease, heart failure, echocardiography, ross score, pediatric cardiology, predictive model

## Abstract

**Highlights:**

**What are the main findings?**

- LV remodelling (LVIDD z-score > +2) and elevated mean pulmonary arterial pressure were the strongest independent echocardiographic predictors of moderate heart failure in pediatric acyanotic CHD, while premature birth showed a paradoxical inverse association.
- An exploratory five-variable model achieved acceptable internally validated discrimination (apparent AUC 0.780; bootstrap-corrected AUC 0.760; LOOCV AUC 0.749), with an optimal MPAP threshold of ≥26.4 mmHg (sensitivity 78.6%).

**What are the implications of the main findings?**

- LVIDD z-score and MPAP may serve as candidate surveillance targets warranting prospective evaluation for identifying children with acyanotic CHD at risk of clinically significant heart failure.
- The inverse association of premature birth warrants prospective investigation, as it may reflect earlier cardiac monitoring in neonatal tertiary settings rather than a true biological advantage.

**Abstract:**

**Background:** Heart failure (HF) remains a major complication of acyanotic congenital heart disease (CHD) in children. Evidence integrating clinical and echocardiographic variables for HF severity stratification in pediatric acyanotic CHD remains limited. This study aimed to identify factors associated with moderate HF and develop an exploratory internally validated prediction model. **Methods**: This retrospective single-center outpatient study included 219 children aged 0–16 years with acyanotic CHD, identified from medical records spanning January 2023 to December 2025. Moderate HF was defined as Ross score 7–9 (≤5 years) or NYHA class III (>5 years). Multivariable analysis was performed using Firth’s penalized logistic regression. Internal validation used bootstrap optimism correction and leave-one-out cross-validation (LOOCV). Model discrimination was assessed using area under the receiver operating characteristic curve (AUC). **Results**: Moderate HF was identified in 131 patients (59.8%). LV remodelling defined by LVIDD z-score > +2 (adjusted OR 3.70, 95% CI 1.22–11.24; *p* = 0.021) and higher mean pulmonary arterial pressure (MPAP) (adjusted OR 1.03 per mmHg, 95% CI 1.00–1.06; *p* = 0.049) were independently associated with moderate HF. Premature birth showed an inverse association with moderate HF (adjusted OR 0.25, 95% CI 0.13–0.48; *p* < 0.001). The exploratory five-variable model demonstrated acceptable discrimination (apparent AUC 0.780, 95% CI 0.728–0.849; bootstrap-corrected AUC 0.760; LOOCV AUC 0.749, 95% CI 0.681–0.811), with adequate calibration. An MPAP threshold of ≥26.4 mmHg yielded 78.6% sensitivity for moderate HF identification. **Conclusions**: LV remodelling and elevated MPAP were independently associated with moderate HF in children with acyanotic CHD. The exploratory internally validated model demonstrated acceptable discrimination using routinely available variables. This model is exploratory and not yet ready for clinical use; prospective multicenter external validation is required before any clinical implementation.

## 1. Introduction

Congenital heart disease (CHD) represents the most common congenital anomaly in children worldwide, with an estimated prevalence of 8–10 per 1000 live births and remains a major contributor to pediatric morbidity and mortality, particularly in low- and middle-income countries [1,2]. Although advances in medical, surgical, and catheter-based interventions have substantially improved survival, heart failure (HF) continues to be a frequent and clinically significant complication, especially during early life [3]. In acyanotic CHD, HF commonly results from chronic left-to-right shunting, pulmonary overcirculation, and progressive cardiac volume overload, leading to ventricular remodelling and pulmonary vascular changes [4,5,6]. Unlike adult HF, pediatric HF is often characterized by preserved systolic function, with symptoms driven primarily by altered loading conditions and limited cardiovascular reserve [7].

Classification is commonly applied in younger children, whereas the New York Heart Association (NYHA) functional classification is more suitable for older children and adolescents [8,9,10]. Beyond structural cardiac abnormalities, HF severity in pediatric CHD is influenced by pulmonary hemodynamics, ventricular remodelling, nutritional status, and the chronicity of hemodynamic burden [11,12,13,14,15,16]. Early identification of children at risk for clinically significant HF is therefore essential to optimize surveillance, guide medical therapy, and support timely intervention. Despite its clinical relevance, nutritional status is often underrepresented in HF risk assessment models. Prediction modelling offers a framework to integrate multiple routinely available variables into a single risk estimate, potentially enabling earlier identification of children at higher risk of clinically meaningful HF [17]. While prediction models have been extensively developed in adult HF populations, pediatric HF remains underrepresented [18,19].

Despite the widespread clinical use of echocardiography, the relative predictive value of individual echocardiographic parameters for HF severity classification in pediatric CHD, particularly according to the Ross Heart Failure Classification Score has not been systematically evaluated using multivariable regression methods. While echocardiographic predictors of functional class have been explored in pediatric cardiomyopathy [20] and machine learning-based phenotyping has been applied to broader pediatric HF cohorts [21], analogous multivariable analyses in acyanotic CHD classified by the Ross Heart Failure Score remain absent.

Understanding which parameters independently predict HF severity would refine echocardiographic interpretation and support evidence-based monitoring thresholds. Identifying clinically meaningful and readily obtainable markers associated with moderate HF may improve outpatient risk stratification and surveillance in children with acyanotic CHD. Therefore, this study aimed to identify clinical and echocardiographic factors associated with moderate HF in pediatric acyanotic CHD and to explore the performance of an internally validated multivariable risk stratification model using routinely available outpatient clinical and echocardiographic variables.

## 2. Materials and Methods

### 2.1. Study Design and Setting

This was a retrospective cohort study conducted at Dr. Soetomo General Hospital, Surabaya, Indonesia. This study included medical records from January 2023 to December 2025. As data were collected retrospectively from existing clinical records, ethics review was sought following the data collection period; data extraction and statistical analysis were performed exclusively after institutional review was completed. This study was conducted in a single tertiary center; potential differences in model performance across socioeconomic or geographic groups were not evaluated. As all predictor variables and HF severity classification were assessed contemporaneously at a single outpatient evaluation, the model functions as a diagnostic severity classification tool rather than a prognostic instrument.

The Health Research Ethics Committee of Dr. Soetomo General Hospital granted exemption from full ethics review for this retrospective study (Letter of Exemption Ref. No. 2314/LOE/301.4.2/III/2026, dated 26 March 2026), conducted in accordance with the Declaration of Helsinki (2013 revision). Informed consent was waived given the retrospective nature of the study and use of anonymized data. Given the retrospective observational design, the study was not prospectively registered, and no formal protocol was prepared before data collection.

### 2.2. Study Population

Consecutive pediatric patients aged 0–16 years with a confirmed diagnosis of acyanotic congenital heart disease (CHD) who underwent transthoracic echocardiographic evaluation in the outpatient pediatric cardiology clinic of a tertiary cardiac center were eligible for enrolment. Patients were excluded if they had: (1) incomplete echocardiographic or medical records; (2) cyanotic congenital heart disease; (3) rheumatic heart disease or other acquired structural cardiac disease; (4) assessment within 30 days after cardiac surgery or catheter intervention; or (5) concurrent non-cardiac systemic illness with potential hemodynamic consequences (e.g., systemic lupus erythematosus or severe anemia). A total of 219 patients fulfilled all eligibility criteria and constituted the final analytical cohort.

### 2.3. Outcome Variable

The primary outcome was moderate heart failure (HF), classified using a two-instrument, age-stratified approach. For patients aged ≤5 years, the Ross Heart Failure Classification Score was applied, a validated, age-adapted instrument analogous to the New York Heart Association (NYHA) functional classification [8,9]. For patients aged >5 years, the modified NYHA classification was used, as recommended for older children [10]. The outcome was dichotomized as: 0 = No HF (Ross 0–2) or Mild HF (Ross 3–6; NYHA class I–II), and 1 = Moderate HF (Ross 7–9; NYHA class III). No patient fulfilled criteria for Severe HF (Ross 10–12; NYHA class IV). HF classification was performed by a pediatric cardiologist blinded to echocardiographic data.

### 2.4. Echocardiographic Assessment

Transthoracic echocardiography was performed by a certified pediatric echocardiographer in accordance with American Society of Echocardiography (ASE) guidelines for pediatric echocardiography [22], with all studies independently reviewed by a senior pediatric cardiologist. Mean pulmonary arterial pressure (MPAP) was estimated using the validated formula MPAP = 0.61 × PSAP + 2 mmHg [23], where pulmonary systolic arterial pressure (PSAP) was derived from the peak tricuspid regurgitation (TR) jet velocity via the simplified Bernoulli equation, with right atrial pressure assumed to be 5 mmHg for all patients, consistent with standard practice in pediatric echocardiographic hemodynamic assessment. TR gradient was measured as the peak instantaneous pressure gradient across the tricuspid valve by continuous-wave Doppler. TR jets were measurable in all 219 patients included in the final cohort; no patients were excluded due to absent or inadequate TR signals. Left ventricular ejection fraction (EF) was quantified by the biplane modified Simpson’s method [22].

Structural defect size for atrial septal defect (ASD), ventricular septal defect (VSD), and patent ductus arteriosus (PDA) was graded as none, small, moderate, or large based on defect dimensions, estimated shunt fraction, and hemodynamic consequences assessed by color and spectral Doppler [24]. Chamber enlargement was recorded as a binary variable if any cardiac chamber dimension exceeded +2 SD above the body surface area-adjusted normative mean (z-score > +2) [25]. Left ventricular remodelling was defined as LV internal dimension in diastole (LVIDD) z-score > +2 using Pettersen et al. normative equations [25] and recorded as a binary variable.

### 2.5. Clinical and Demographic Variables

The following clinical covariates were recorded. Age was recorded as a continuous variable in years at the time of assessment, and sex as female or male. Premature birth was dichotomized as yes or no based on gestational age < 37 weeks at birth as documented in the medical record. Nutritional status was classified on a four-level ordinal scale (normal, mild wasting, moderate wasting, severe wasting) using weight-for-height z-scores according to WHO Child Growth Standards [26]. Age at CHD diagnosis was dichotomized as ≤1 year or >1 year, used as a proxy for cumulative hemodynamic burden duration. Sildenafil use was dichotomized as short-term (<3 months) or sustained (≥3 months) exposure to phosphodiesterase type-5 inhibitor therapy for pulmonary arterial hypertension.

### 2.6. Statistical Analysis

This study was conducted and reported in accordance with the Transparent Reporting of a Multivariable Prediction Model for Individual Prognosis or Diagnosis (TRIPOD+AI) statement [27]. The completed TRIPOD+AI checklist is provided as Supplementary Material. All 219 patients included in the final analytical cohort had complete data for all candidate predictors and the primary outcome. No imputation or other missing data handling procedures were therefore required.

#### 2.6.1. Descriptive Statistics and Group Comparisons

Continuous variables are presented as median (interquartile range [IQR]) and compared between groups using the Mann–Whitney U test (normal approximation with continuity correction). Categorical variables are expressed as frequency and percentage [n (%)] and compared using Fisher’s exact test (2 × 2 tables) or Pearson’s chi-square test (k × 2 tables; k > 2). Statistical significance was defined as *p* < 0.05 (two-sided).

#### 2.6.2. Univariate Logistic Regression

Univariate binary logistic regression using Firth’s penalized likelihood method was performed for each candidate predictor against the dichotomous outcome (moderate HF vs. no/mild HF). Results are expressed as unadjusted odds ratios (OR) with 95% Wald confidence intervals (CI). Variables meeting a pre-specified screening threshold of *p* < 0.20 were considered candidates for multivariable inclusion [28].

#### 2.6.3. Collinearity Assessment

Pairwise Pearson correlations and variance inflation factors (VIF) were computed for all continuous candidate predictors. Full pairwise Pearson correlation coefficients are provided in Supplementary Table S1. MPAP, PSAP, and TR gradient demonstrated near-perfect collinearity (all r > 0.99), consistent with their mathematical interdependence. Only MPAP was retained in multivariable models as the primary hemodynamic predictor. A VIF threshold of 5.0 was applied; all retained predictors met this criterion.

#### 2.6.4. Multivariable Logistic Regression

Multivariable logistic regression was fitted using Firth’s penalized likelihood method (Jeffreys invariant prior penalty), which provides bias-reduced parameter estimates and is specifically recommended for settings with small event counts or near-complete separation. No class imbalance correction methods were applied, as Firth’s penalized regression inherently addresses near-separation issues and the outcome ratio (40:60) was not considered clinically extreme.

Sample size adequacy was evaluated according to the events-per-variable (EPV) principle for logistic regression modelling. With 131 outcome events and five predictors in the primary model, the study achieved an EPV of 26.2, substantially exceeding the conventional minimum threshold of 10 EPV recommended to reduce overfitting and ensure stable coefficient estimation [29]. The extended eight-variable model achieved EPV = 16.4. Two pre-specified models were evaluated:

Exploratory five-variable model: Premature birth + nutritional status + MPAP + chamber enlargement + LV remodelling. Selected a priori based on clinical plausibility (covering patient vulnerability, hemodynamic burden, and structural cardiac remodelling domains), univariate significance, and acceptable EPV.

Extended eight-variable model: All 8 candidates meeting *p* < 0.20 threshold from univariate analysis: MPAP, age, chamber enlargement, nutritional status, premature birth, age at CHD diagnosis, sildenafil use, and LV remodelling. Pre-specified to assess incremental discriminative value over the exploratory five-variable model.

#### 2.6.5. Model Discrimination and Calibration

Discriminative performance was assessed by the area under the receiver operating characteristic curve (AUC; equivalent to the C-statistic). AUC values of 0.70–0.80 were considered acceptable, 0.80–0.90 excellent, and >0.90 outstanding [28]. The optimal MPAP threshold was identified by Youden’s J index (J = Sensitivity + Specificity − 1) [30], which maximizes the joint sensitivity and specificity without imposing a priori differential weighting on false-positive or false-negative misclassification, an approach appropriate for exploratory outpatient surveillance contexts where no established clinical threshold for misclassification costs exists. Internal validity was assessed using: (1) Harrell’s bootstrap optimism correction (B = 1000 resamples), subtracting mean optimism from the apparent AUC; and (2) leave-one-out cross-validation (LOOCV).

Model calibration was primarily assessed using calibration slope and intercept for apparent, bootstrap-corrected, and LOOCV-validated estimates, where a slope of 1.0 and intercept of 0 indicate perfect calibration. Overall predictive accuracy was quantified using the Brier score, with the Brier Skill Score (BSS) calculated relative to a null model. Calibration was visualized by plotting mean predicted probabilities against observed event rates across deciles with 95% confidence intervals, for both apparent and LOOCV-validated estimates. The Hosmer-Lemeshow goodness-of-fit test (10 deciles, df = 8) was additionally performed as a supplementary measure, acknowledging its known dependence on sample size.

#### 2.6.6. Sensitivity Analysis

As a sensitivity analysis, the exploratory five-variable model was re-fitted in children aged ≤5 years (n = 204) to assess whether model discrimination was materially influenced by the near-complete outcome separation observed in children aged >5 years, in whom all cases were classified as moderate HF by NYHA criteria.

#### 2.6.7. Software

All statistical analyses were conducted in Python (version 3.11), using SciPy (version 1.11) and scikit-learn (version 1.3). Firth’s penalized logistic regression was implemented using a custom iteratively reweighted least squares (IRLS) routine with Jeffreys invariant prior penalty. Bootstrap optimism correction (B = 1000 resamples) and leave-one-out cross-validation were implemented using custom NumPy-based routines.

## 3. Results

**Figure 1:**
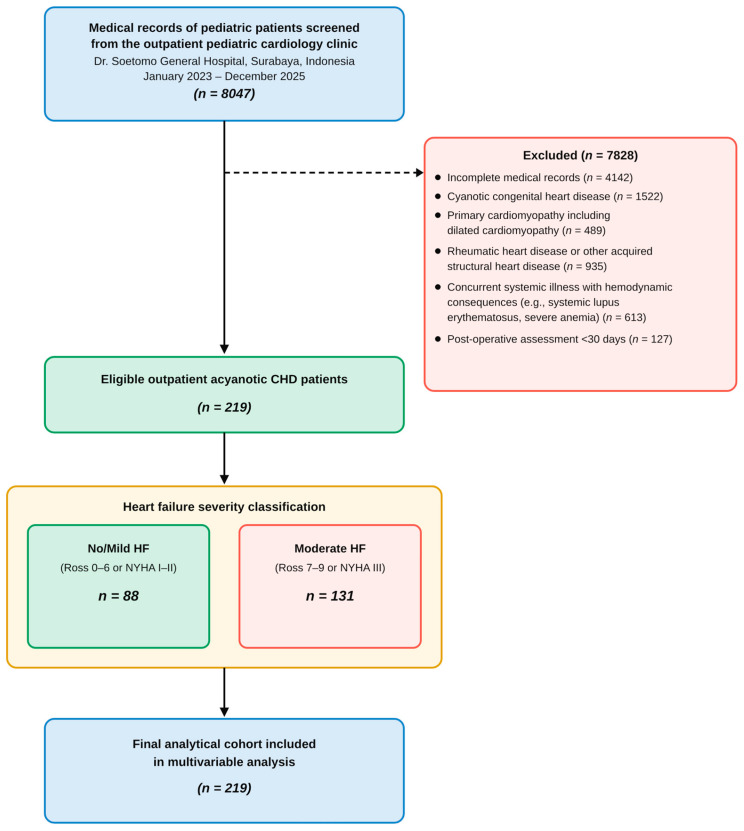
Participant selection flowchart. CHD = congenital heart disease; HF = heart failure; NYHA = New York Heart Association; Ross = Ross Heart Failure Classification. Solid arrows represent the sequential flow of patients through eligibility criteria; the dashed arrow indicates patients excluded from the study cohort.

Of 8047 medical records screened, 219 patients met the eligibility criteria and were included in the final analytical cohort (Figure 1). Of these, 88 (40.2%) were classified as No/Mild HF and 131 (59.8%) as Moderate HF.

### 3.1. Study Population and Baseline Characteristics

A total of 219 pediatric patients with acyanotic CHD were included. The median age was 2.0 years (IQR 1.5–3.8); 123 patients (56.2%) were female. Of the total cohort, 81 patients (37.0%) were born prematurely. The majority (78.1%) had a CHD diagnosis established more than one year before assessment. Based on the age-stratified HF classification, 88 patients (40.2%) were classified as no/mild HF and 131 (59.8%) as moderate HF. All patients aged >5 years (n = 15) fulfilled criteria for moderate HF based on the NYHA classification, producing near-complete separation in age-stratified analyses.

Patients with moderate HF demonstrated significantly higher pulmonary arterial pressures: median MPAP 31.3 (IQR 26.7–38.3) vs. 26.9 (IQR 20.2–34.0) mmHg (*p* = 0.0006). Premature birth was markedly more prevalent in the no/mild HF group (56.8% vs. 23.7%, *p* < 0.001). Chamber enlargement was present in 83.2% of moderate vs. 56.8% of no/mild HF patients (*p* < 0.001), and LV remodelling in 93.9% vs. 75.0% (*p* < 0.001). Nutritional status differed significantly between groups (*p* < 0.001), with moderate-to-severe wasting predominant in both groups but normal nutritional status virtually absent in the moderate HF group (0.8% vs. 26.1%). The distribution of CHD type did not differ significantly between groups (*p* = 0.391), with ASD and VSD being the most common lesions in both no/mild HF (40.9% and 35.2%, respectively) and moderate HF (39.7% and 38.9%, respectively). However, defect size distributions differed significantly for ASD (*p* = 0.002) and VSD (*p* < 0.001), with large defects more prevalent in the moderate HF group (ASD: 37.4% vs. 25.0%; VSD: 13.0% vs. 0.0%). Ejection fraction, sex, and PDA defect size did not differ significantly between groups. Full baseline characteristics are presented in Table 1.

### 3.2. Univariate Logistic Regression

Univariate Firth’s logistic regression identified nine variables significantly associated with moderate HF (all *p* < 0.05): Age (OR 1.292; 95% CI 1.095–1.525; *p* = 0.002), TR Gradient (OR 1.022; *p* = 0.007), PSAP (OR 1.021; *p* = 0.011), MPAP (OR 1.038; *p* = 0.005), Chamber Enlargement (OR 3.753; 95% CI 2.014–6.994; *p* < 0.001), Nutritional Status (OR 2.005; 95% CI 1.414–2.843; *p* < 0.001), Premature Birth (OR 0.236; 95% CI 0.132–0.423; *p* < 0.001), Sildenafil ≥ 3 months (OR 2.298; 95% CI 1.302–4.056; *p* = 0.004), and LV Remodelling (OR 5.081; 95% CI 2.147–12.024; *p* < 0.001). Premature Birth was the only variable with an OR below unity, indicating an inverse association. EF, sex, ASD size, VSD size, and PDA size were not significantly associated with moderate HF. Age at CHD Diagnosis met the *p* < 0.20 screening threshold (*p* = 0.119). Although mixed lesion type (≥2 lesions) also met the *p* < 0.20 threshold (OR 0.424; *p* = 0.116), it was not carried forward to multivariable modelling due to small cell frequency (n = 15) and limited clinical interpretability as an independent predictor. PSAP and TR Gradient were excluded from multivariable modelling due to near-perfect collinearity with MPAP (r > 0.99). The significant distributional differences observed for ASD and VSD size in bivariate comparisons (Table 1) reflect non-monotonic distributions across categories rather than a consistent directional trend, accounting for their non-significance in ordinal logistic regression. Full results are presented in Table 2.

### 3.3. Multivariable Logistic Regression

In the exploratory five-variable model, three variables were independently and significantly associated with moderate HF: premature birth (adjusted OR 0.245; 95% CI 0.125–0.479; *p* < 0.001), MPAP (adjusted OR 1.030; 95% CI 1.000–1.061; *p* = 0.049), and LV remodelling (adjusted OR 3.703; 95% CI 1.220–11.237; *p* = 0.021). Nutritional status (adjusted OR 1.374; *p* = 0.126) and chamber enlargement (adjusted OR 1.875; *p* = 0.107) showed directionally consistent trends but did not reach statistical significance in the adjusted model. Multicollinearity was negligible across all five predictors (VIF range 1.149–1.512, all well below the threshold of 5.0). The model demonstrated acceptable discrimination (AUC = 0.780). Bootstrap optimism correction (B = 1000 resamples; Harrell’s method) yielded a mean optimism of 0.021, giving a corrected AUC of 0.760. LOOCV produced a consistent, conservative estimate (LOOCV AUC = 0.749).

The extended eight-variable model yielded only marginal improvement over the exploratory five-variable model (apparent AUC = 0.797; LOOCV AUC = 0.749), confirming no additional predictive value of the three extra variables (age, age at CHD diagnosis, sildenafil use) beyond those in the exploratory five-variable model. In the extended eight-variable model, premature birth (adjusted OR 0.273; *p* < 0.001) and LV remodelling (adjusted OR 4.395; *p* = 0.014) remained the sole statistically significant independent predictors, with MPAP attenuated to non-significance (*p* = 0.146), likely reflecting confounding by age. Multivariable results are presented in Table 3.

The apparent AUC gain of the extended model was modest (+0.017), and this improvement was not sustained under internal validation, with LOOCV AUC identical to the five-variable model (0.749 vs. 0.749; ΔAUC = 0). The apparent-to-LOOCV shrinkage was also greater in the extended model (Δ = 0.048) than in the five-variable model (Δ = 0.031), suggesting greater optimism associated with the three additional predictors. Given equivalent internally validated discrimination, a lower EPV (16.4 vs. 26.2), and the added interpretability and data requirements of the simpler model, the five-variable model was selected as the preferred candidate for prospective external validation.

To facilitate individual risk estimation and future external validation, the full regression equation of the exploratory five-variable model is provided below:

logit(P)=−2.090−1.406(Premature Birth)+0.318(Nutritional Status)   +0.030(MPAP)+0.629(Chamber Enlargement)+1.309(LV Remodelling)          

where premature birth, chamber enlargement, and LV remodelling were coded as binary variables (1 = present, 0 = absent), Nutritional Status was coded as an ordinal variable (0 = normal, 1 = mild wasting, 2 = moderate wasting, 3 = severe wasting), and MPAP was entered continuously in mmHg.

The predicted probability of moderate HF was calculated as:

$$P=\frac{1}{1+e^{-logit(P)}}$$

### 3.4. Model Discrimination and Optimal MPAP Cutpoint

**Figure 2:**
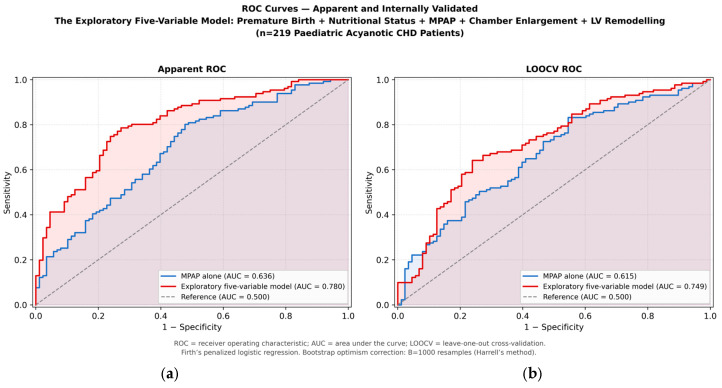
ROC curves for MPAP alone and the exploratory five-variable model (Premature Birth + Nutritional Status + MPAP + Chamber Enlargement + LV Remodelling). (**a**) apparent ROC; (**b**) leave-one-out cross-validated (LOOCV) ROC. Bootstrap-corrected AUC values are annotated on the apparent panel. ROC = receiver operating characteristic; AUC = area under the curve; LOOCV = leave-one-out cross-validation; MPAP = mean pulmonary arterial pressure; CHD = congenital heart disease. Firth’s penalized logistic regression throughout.

ROC curves for MPAP alone and the exploratory five-variable model, apparent and internally validated, are depicted in Figure 2. The exploratory five-variable model demonstrated markedly superior discrimination compared with MPAP alone (apparent AUC 0.780, 95% CI 0.728–0.849 vs. 0.636; ΔAUC = +0.144). After bootstrap optimism correction, the exploratory five-variable model retained acceptable discrimination (corrected AUC 0.760 vs. 0.639; corrected ΔAUC = +0.121). LOOCV confirmed this pattern (LOOCV AUC 0.749, 95% CI 0.681–0.811 vs. 0.615; ΔAUC = +0.134). Full bootstrap optimism correction details are provided in Supplementary Table S2. ROC curve comparisons between both models are presented in Supplementary Figure S1.

Using Youden’s J index, an MPAP threshold of ≥26.4 mmHg yielded the optimal cutpoint for identifying moderate HF (Sensitivity = 78.6%; Specificity = 47.7%; Youden J = 0.264). In the sensitivity analysis restricted to children aged ≤5 years (n = 204; 116 moderate HF events), the model retained acceptable discrimination (apparent AUC 0.762, 95% CI 0.697–0.826; bootstrap-corrected AUC 0.741; LOOCV AUC 0.727, 95% CI 0.659–0.795), consistent with the primary analysis (Table S3).

### 3.5. Model Calibration

**Figure 3:**
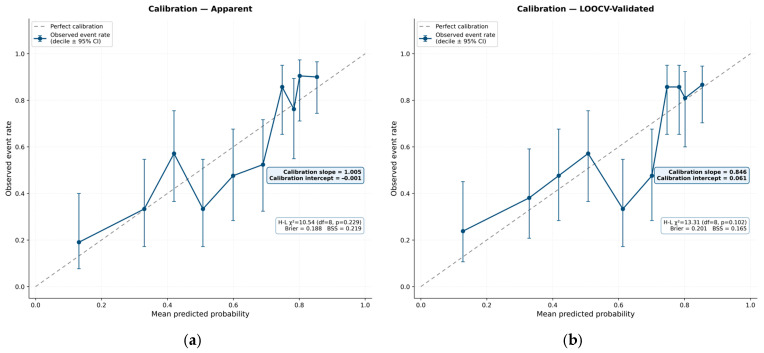
Calibration plots for the exploratory five-variable Firth’s logistic regression model (Premature Birth + Nutritional Status + MPAP + Chamber Enlargement + LV Remodelling). (**a**) Apparent calibration; (**b**) LOOCV-validated calibration. Each point represents the mean predicted probability versus observed event rate within a predicted probability decile (error bars: 95% Wilson confidence interval). The dashed diagonal line indicates perfect calibration. Calibration slope and intercept are reported within each panel as primary measures of calibration agreement. H-L = Hosmer-Lemeshow; BSS = Brier Skill Score; LOOCV = leave-one-out cross-validation.

Model calibration was primarily assessed using calibration slope and intercept, graphical calibration plots, and the Brier score. The apparent calibration slope was 1.005 (intercept −0.001), indicating excellent apparent calibration with no systematic over- or under-prediction. After bootstrap optimism correction, the calibration slope was 0.896 (intercept 0.030), and LOOCV-validated calibration yielded a slope of 0.846 (intercept 0.061), both indicating acceptable calibration with minimal optimism. The apparent Brier score was 0.188 (Brier Skill Score = 0.219), with a LOOCV-corrected estimate of 0.201 (BSS = 0.165). The Hosmer-Lemeshow goodness-of-fit test, reported as a supplementary measure, showed no significant lack of fit in either the apparent (χ^2^ = 10.54, df = 8, *p* = 0.229) or LOOCV-validated (χ^2^ = 13.31, df = 8, *p* = 0.102) samples; however, this test is acknowledged to be sample-size dependent and was not used as the primary calibration criterion. Calibration plots for apparent and LOOCV-validated predictions are presented in Figure 3.

## 4. Discussion

This retrospective study identified LV remodelling, elevated pulmonary arterial pressure, and premature birth as variables independently associated with moderate HF severity in children with acyanotic CHD. An exploratory multivariable model demonstrated adequate internally validated discrimination. These findings support a hypothesis-generating framework for echocardiographic HF risk stratification in pediatric acyanotic CHD.

Echocardiography represents the primary imaging modality for diagnosis and ongoing monitoring in CHD across different life stages [31]. The present study leverages this established role by utilizing routinely obtained echocardiographic parameters as candidate markers for HF severity stratification in pediatric acyanotic CHD. From an anatomical perspective, acyanotic CHD lesions characterized by chronic left-to-right shunting are known to impose sustained volume overload on the left heart, contributing to progressive LV dilation and ventricular remodelling [14]. The emergence of LV remodelling as the strongest structural correlate of moderate HF in the present cohort may be explained, at least in part, by the cumulative hemodynamic burden imposed by prolonged volume overload in acyanotic CHD. Elevated LVIDD z-scores may therefore reflect not only the anatomical consequence of left-to-right shunting, but also the degree to which the ventricle has adapted to sustained overcirculation, though this interpretation requires longitudinal validation. These observations are supported by prior evidence demonstrating that LV dilation in pediatric volume-overload CHD may occur despite preserved systolic function [32,33], and that ventricular dimension z-scores track with clinical HF severity in children with CHD receiving neurohormonal blockade [34]. Interestingly, EF was not independently associated with moderate HF. This finding is consistent with the recognized pathophysiology of over-circulation heart failure in children, in which volume overload of cardiac chambers occurs in the presence of normal or even hypercontractile LV function [33]. Corroborating this, a recent institutional study documented preserved ejection fraction (mean 75.9 ± 9.1%) across all children with acyanotic CHD and clinical heart failure at the same centre [35]. In the context of acyanotic CHD with volume overload, altered loading conditions may limit the ability of conventional systolic indices such as EF to fully reflect the degree of hemodynamic compromise.

Hemodynamically, elevated MPAP was independently associated with increased odds of moderate HF after adjustment for other covariates. Chronic pulmonary overcirculation in acyanotic CHD contributes not only to ventricular volume overload but also to progressive pulmonary vascular remodelling and increasing pulmonary arterial pressure [14]. The finding that each 1-mmHg increase in MPAP was associated with approximately 3% greater odds of moderate HF is clinically relevant given the relatively narrow pressure range observed in this outpatient cohort. The optimal MPAP cut-point derived from ROC analysis (≥26.4 mmHg, Youden’s J index) closely approximates the hemodynamic definition of pulmonary hypertension in children (MPAP > 25 mmHg) as established by the AHA/ATS Pediatric Pulmonary Hypertension Guidelines [36], supporting its physiologic plausibility. However, the relatively modest specificity reflects the inherent trade-off of Youden’s index: prioritizing sensitivity over specificity, a trade-off acceptable in outpatient surveillance but reinforcing that MPAP elevation alone is insufficient for definitive HF severity classification. Consistent with this, defect size itself was not independently associated with HF severity after regression adjustment, suggesting that downstream hemodynamic and remodelling consequences are more clinically relevant determinants than anatomical dimensions alone.

The outpatient-based nature of this cohort has important implications for the observed HF severity distribution. The high proportion of moderate HF (59.8%) likely reflects a selection effect inherent to tertiary outpatient settings: patients with no or mild HF may not be referred, while those with severe decompensated HF are typically managed in inpatient settings. This cohort therefore represents a clinically intermediate stratum that may not reflect the full spectrum of HF severity in pediatric acyanotic CHD, and should be considered when interpreting the generalizability of model findings. This outpatient selection pattern may also explain the primacy of chronic hemodynamic burden markers over overt systolic dysfunction as correlates of HF severity in this cohort. The high prevalence of nutritional compromise in this cohort is consistent with prior institutional data, where the majority of acyanotic CHD patients exhibited underweight or undernutrition across age groups [37,38]. Nutritional status demonstrated clinically meaningful trends despite not reaching statistical significance in the adjusted model, possibly reflecting collinearity with chronic remodelling variables [15,16].

The inverse association observed with premature birth warrants cautious interpretation and should not be construed as a protective biological effect. Two structural biases are likely to substantially contribute to this finding. First, referral bias: premature infants in tertiary neonatal care settings typically undergo earlier and more frequent echocardiographic evaluation, resulting in earlier CHD detection and intervention before progression to moderate HF [39]. Second, survivorship bias: extremely premature infants with severe hemodynamic compromise may not survive to outpatient evaluation, systematically excluding the most severely affected from this cohort. Together, these biases may preferentially enrich the premature birth group with clinically milder cases, producing an apparent inverse association that reflects care pathways rather than disease biology. Residual confounding by unmeasured variables, including gestational age, birth weight, and neonatal intervention history, cannot be excluded. This finding should therefore be considered hypothesis-generating and requires prospective validation with detailed gestational-age stratification and neonatal intervention data.

The exploratory five-variable model demonstrated acceptable internally validated discrimination, with bootstrap-corrected and LOOCV AUC values comparable to previously published regression-based pediatric cardiac prediction models [40,41,42]. Unlike several prior models that focused primarily on perioperative outcomes or disease-specific prognostication [41,42], the present model specifically targeted clinically significant HF in children with acyanotic CHD using routinely obtainable echocardiographic and clinical variables. Importantly, the more complex extended eight-variable model did not improve LOOCV performance (ΔAUC = 0), while exhibiting greater apparent-to-validated shrinkage (Δ = 0.048 vs. Δ = 0.031) and a lower EPV (16.4 vs. 26.2), suggesting greater optimism associated with the additional predictors. In clinical prediction modelling, a simpler model is preferred when validated discrimination is equivalent, as parsimony reduces overfitting risk, enhances interpretability, and imposes fewer data requirements for external validation [17]. Although machine learning approaches have demonstrated higher discriminative performance in some pediatric cardiac populations [40], regression-based models maintain advantages in interpretability and transparency, which are particularly important for hypothesis-generating models in outpatient and resource-limited settings. When MPAP estimation is not feasible due to absent or suboptimal tricuspid regurgitation signals, the hemodynamic component may be compromised and predictions should be interpreted cautiously.

Several limitations warrant acknowledgement. First, HF classification employed two instruments (Ross Score for ≤5 years, NYHA for >5 years), which were combined into a single binary outcome variable. This approach assumes functional equivalence between the two instruments at the “moderate” threshold, an assumption that may not be fully justified, as the Ross Score incorporates objective physiological parameters (feeding behaviour, respiratory rate, growth, and peripheral perfusion), whereas the NYHA classification relies primarily on subjective functional capacity assessment. Systematic differences in sensitivity and specificity between these instruments may therefore introduce classification heterogeneity that could differentially affect predictor–outcome associations across age groups, potentially biasing model estimates in ways that cannot be fully corrected by statistical methods. Furthermore, all 15 patients aged >5 years were classified as moderate HF, producing near-complete outcome separation that may have introduced estimation instability. Firth’s penalized regression was applied to mitigate this, and a sensitivity analysis restricted to children aged ≤5 years (n = 204) confirmed consistent model discrimination (LOOCV AUC 0.727), suggesting that model performance was not materially driven by this subgroup. A fully unified classification instrument would strengthen future studies. Second, the retrospective design precludes causal inference and is susceptible to residual confounding by unmeasured variables such as BNP/NT-proBNP, oxygen saturation, or exact CHD lesion subtype. Furthermore, right ventricular function parameters such as tricuspid annular plane systolic excursion (TAPSE) were not systematically assessed; prior work from our group demonstrated that TAPSE is significantly reduced in the presence of hemodynamic stress and may provide incremental prognostic information beyond left ventricular ejection fraction [43], representing a candidate predictor for future prospective studies. Third, 7828 of 8047 screened records were excluded due to incomplete data; as characteristics of excluded patients could not be ascertained, systematic differences between included and excluded patients cannot be ruled out, potentially limiting generalizability. Finally, as predictor variables and outcome were assessed contemporaneously, this prediction model functions as a diagnostic severity classification tool rather than a prognostic instrument. Collectively, these limitations underscore the need for prospective multicenter external validation with a more balanced age distribution and formal subgroup analyses across sex, age, and CHD lesion type before any clinical application can be considered.

## 5. Conclusions

LV remodelling and elevated pulmonary arterial pressure were independently associated with moderate heart failure severity in children with acyanotic congenital heart disease, while premature birth demonstrated an inverse association that may reflect earlier surveillance or referral patterns. An exploratory multivariable model demonstrated acceptable internally validated discrimination (bootstrap-corrected AUC 0.760; LOOCV AUC 0.749) using routinely available outpatient variables; however, these hypothesis-generating findings should be interpreted cautiously given the retrospective single-center design and absence of external validation. Prospective multicenter studies are required to evaluate the generalizability and clinical utility of this prediction model.

## Figures and Tables

**Table 1:** Baseline Characteristics by Heart Failure Severity.

Variable	No/Mild HF (n = 88)	Moderate HF (n = 131)	*p*-Value	Test
Continuous Variables—Median (IQR)				
Age (years)	2.0 (1.0–3.0)	2.5 (1.6–3.9)	0.0039 *	MWU
TR Gradient (mmHg)	41.5 (30.0–52.5)	48.0 (40.5–59.5)	0.0011 *	MWU
PSAP (mmHg)	46.5 (35.0–58.2)	53.0 (45.0–64.5)	0.0019 *	MWU
MPAP (mmHg)	26.9 (20.2–34.0)	31.3 (26.7–38.3)	0.0006 *	MWU
Ejection Fraction (%)	74.4 (69.0–81.8)	76.0 (70.3–81.0)	0.389	MWU
Categorical Variables—n (%)				
Female sex	47 (53.4%)	76 (58.0%)	0.579	Fisher
Premature birth	50 (56.8%)	31 (23.7%)	<0.001 *	Fisher
Age at CHD Diagnosis > 1 year	64 (72.7%)	107 (81.7%)	0.135	Fisher
Sildenafil ≥ 3 months	47 (53.4%)	95 (72.5%)	0.004 *	Fisher
Chamber Enlargement	50 (56.8%)	109 (83.2%)	<0.001 *	Fisher
LV Remodelling (LVIDD z > +2)	66 (75.0%)	123 (93.9%)	<0.001 *	Fisher
Nutritional Status—n (%)				
Normal	23 (26.1%)	1 (0.8%)	<0.001 *	Chi^2^
Mild wasting	20 (22.7%)	38 (29.0%)	—	—
Moderate wasting	35 (39.8%)	74 (56.5%)	—	—
Severe wasting	10 (11.4%)	18 (13.7%)	—	—
CHD Type—n (%)				
ASD	36 (40.9%)	52 (39.7%)	0.3915	Chi^2^
VSD	31 (35.2%)	51 (38.9%)		
PDA	12 (13.6%)	22 (16.8%)		
Mixed (≥2 lesions)	9 (10.2%)	6 (4.6%)		
ASD Defect Size—n (%)				
None	44 (50.0%)	73 (55.7%)	0.002 *	Chi^2^
Small	11 (12.5%)	5 (3.8%)	—	—
Moderate	11 (12.5%)	4 (3.1%)	—	—
Large	22 (25.0%)	49 (37.4%)	—	—
VSD Defect Size—n (%)				
None	49 (55.7%)	75 (57.3%)	<0.001 *	Chi^2^
Small	10 (11.4%)	4 (3.1%)	—	—
Moderate	29 (33.0%)	35 (26.7%)	—	—
Large	0 (0.0%)	17 (13.0%)	—	—
PDA Defect Size—n (%)				
None	73 (83.0%)	108 (82.4%)	0.3535	Chi^2^
Small	8 (9.1%)	6 (4.6%)		
Moderate	7 (8.0%)	16 (12.2%)		
Large	0 (0.0%)	1 (0.8%)		

* *p* < 0.05. MWU = Mann–Whitney U test. IQR = interquartile range. MPAP = mean pulmonary arterial pressure. PSAP = pulmonary systolic arterial pressure. TR = tricuspid regurgitation. ASD = atrial septal defect. VSD = ventricular septal defect. PDA = patent ductus arteriosus. LV = left ventricular. LVIDD = LV internal dimension in diastole. CHD = congenital heart disease. HF = heart failure. Chi^2^ = chi-square test.

**Table 2:** Univariate Firth’s Logistic Regression.

Variable	OR	95% CI (Wald)	*p*-Value	Notes
Age (years)	1.292	1.095–1.525	0.002 *	
Sex (male)	0.830	0.482–1.429	0.501	
Nutritional Status (ordinal)	2.005	1.414–2.843	<0.001 *	
Premature Birth	0.236	0.132–0.423	<0.001 *	Inverse association (OR < 1)
Age at CHD Dx > 1 yr	1.671	0.877–3.184	0.119	‡ Candidate (*p* < 0.20)
Sildenafil ≥ 3 months	2.298	1.302–4.056	0.004 *	
CHD Type (Reference: ASD only)				
VSD only	1.171	0.668–2.052	0.581	
PDA only	1.273	0.595–2.727	0.534	
Mixed (≥2 lesions)	0.424	0.146–1.237	0.116	§ Excluded from MV (small cell frequency)
ASD Defect Size (ordinal)	1.053	0.863–1.284	0.611	
VSD Defect Size (ordinal)	1.172	0.908–1.513	0.224	
PDA Defect Size (ordinal)	1.154	0.761–1.751	0.500	
TR Gradient (mmHg)	1.022	1.006–1.038	0.007 *	† Excluded from MV (collinear)
PSAP (mmHg)	1.021	1.005–1.037	0.011 *	† Excluded from MV (collinear)
MPAP (mmHg)	1.038	1.012–1.066	0.005 *	Retained as hemodynamic predictor
Ejection Fraction (%)	1.013	0.980–1.048	0.435	
Chamber Enlargement	3.753	2.014–6.994	<0.001 *	
LV Remodelling (LVIDD)	5.081	2.147–12.024	<0.001 *	Strongest structural predictor

* *p* < 0.05. OR = unadjusted odds ratio; CI = 95% Wald confidence interval. Firth’s penalized logistic regression. † Excluded from multivariable modelling due to collinearity with MPAP (r > 0.99). ‡ *p* < 0.20—candidate for multivariable inclusion. § Small cell frequency (n = 15); excluded from multivariable modelling. MPAP = mean pulmonary arterial pressure. PSAP = pulmonary systolic arterial pressure. TR = tricuspid regurgitation. LV = left ventricular. LVIDD = LV internal dimension in diastole.

**Table 3:** Multivariable Firth’s Logistic Regression.

Variable	Adj. OR	95% CI	*p*-Value	Notes
EXPLORATORY FIVE-VARIABLE MODEL: Premature + Nutritional Status + MPAP + Chamber Enlargement + LV Remodelling (EPV = 26.2)				
Premature Birth	0.245	0.125–0.479	<0.001 *	Strongest independent predictor
Nutritional Status	1.374	0.915–2.064	0.126	Trend; *p* < 0.20
MPAP (mmHg)	1.030	1.000–1.061	0.049 *	Each 1 mmHg ~3% greater odds
Chamber Enlargement	1.875	0.874–4.024	0.107	Trend; *p* < 0.20
LV Remodelling (LVIDD)	3.703	1.220–11.237	0.021 *	~3.7× greater odds of moderate HF
Model Performance				
Apparent AUC	0.780	0.728–0.849		
Bootstrap-corrected AUC (B = 1000)	0.760			Optimism = 0.021 (Harrell’s method)
LOOCV AUC	0.749	0.681–0.811		Conservative internal validation estimate
Optimal MPAP cutpoint (Youden’s J)	≥26.4 mmHg			Sensitivity 78.6%; Specificity 47.7%; J = 0.264
EXTENDED EIGHT-VARIABLE MODEL: All 8 candidates (*p* < 0.20 from univariate; EPV = 16.4)				
Apparent AUC	0.797			Marginal gain over five-variable model (+0.017 LOOCV)
Bootstrap-corrected AUC (B = 1000)	0.762			
LOOCV AUC	0.749			No improvement over five-variable model (ΔAUC = 0)

* *p* < 0.05. Adj. OR = adjusted odds ratio; CI = 95% Wald CI; EPV = events per variable (based on n = 131 moderate HF outcome events); AUC = area under ROC curve; LOOCV = leave-one-out cross-validation. Firth’s penalized logistic regression throughout. Bootstrap optimism correction: B = 1000 resamples, Harrell’s method. MPAP = mean pulmonary arterial pressure; LV = left ventricular; LVIDD = LV internal dimension in diastole. All retained predictors had VIF < 2.0. Individual predictor estimates for the extended eight-variable model are reported in Supplementary Table S4.

## Data Availability

The data presented in this study are available on request from the corresponding author due to patient privacy and institutional data governance policies of Dr. Soetomo General Hospital, Surabaya, Indonesia. The analytical code supporting the findings of this study is also available from the corresponding author upon reasonable request.

## Supplementary Materials

The following supporting information can be downloaded at: https://www.mdpi.com/article/10.3390/children13060809/s1, Table S1: Pearson Correlation Matrix—Continuous Candidate Predictors; Table S2: Bootstrap Optimism Correction Summary (B = 1000 Resamples, Harrell’s Method); Table S3: Sensitivity Analysis—Exploratory Five-Variable Model Performance Restricted to Children Aged ≤5 Years; Table S4: Extended Eight-Variable Model—Individual Predictor Estimates (Firth’s Penalized Logistic Regression; EPV = 16.4); Figure S1: ROC Curves (Apparent and LOOCV)—Exploratory Five-Variable Model vs. Extended Eight-Variable Model; File S1: Completed TRIPOD+AI Checklist.

## Abbreviations

The following abbreviations are used in this manuscript:

ASD	Atrial Septal Defect
ASE	American Society of Echocardiography
AUC	Area Under the Curve
BNP	B-type Natriuretic Peptide
BSS	Brier Skill Score
CHD	Congenital Heart Disease
CI	Confidence Interval
EF	Ejection Fraction
EPV	Events Per Variable
HF	Heart Failure
IQR	Interquartile Range
IRLS	Iteratively Reweighted Least Squares
LOOCV	Leave-One-Out Cross-Validation
LV	Left Ventricular
LVIDD	Left Ventricular Internal Dimension in Diastole
MPAP	Mean Pulmonary Arterial Pressure
MWU	Mann–Whitney U Test
NT-proBNP	N-terminal pro-B-type Natriuretic Peptide
NYHA	New York Heart Association
OR	Odds Ratio
PAH	Pulmonary Arterial Hypertension
PDA	Patent Ductus Arteriosus
PH	Pulmonary Hypertension
PSAP	Pulmonary Systolic Arterial Pressure
ROC	Receiver Operating Characteristic
SD	Standard Deviation
TAPSE	Tricuspid annular plane systolic excursion
TR	Tricuspid Regurgitation
VIF	Variance Inflation Factor
VSD	Ventricular Septal Defect
WHO	World Health Organization
